# Multidrug resistance in *Helicobacter pylori* infection

**DOI:** 10.3389/fmicb.2023.1128497

**Published:** 2023-02-27

**Authors:** Raluca Ioana Dascălu, Alexandra Bolocan, Dan Nicolae Păduaru, Alexandru Constantinescu, Magda Mihaela Mitache, Anca Daniela Stoica, Octavian Andronic

**Affiliations:** ^1^Emergency Clinical Hospital of Bucharest, Bucharest, Romania; ^2^Carol Davila University of Medicine and Pharmacy, Bucharest, Romania; ^3^University Emergency Hospital of Bucharest, Bucharest, Romania; ^4^Titu Maiorescu University, Bucharest, Romania; ^5^Babeş-Bolyai University, Cluj-Napoca, Romania; ^6^National Institute for Research and Development of Isotopic and Molecular Technologies, Cluj-Napoca, Romania; ^7^Research Institute of the University of Bucharest, Bucharest, Romania

**Keywords:** *Helicobacter pylori*, infection, multidrug resistance, antibiotic, eradication

## Abstract

*Helicobacter pylori* (Hp), a well-known human pathogen, causes one of the most common chronic bacterial infections and plays an important role in the emergence of chronic progressive gastric inflammation and a variety of gastrointestinal diseases. The prevalence of Hp infection varies worldwide and is indirectly proportional to socio-economic status, especially during childhood. The response to the eradication therapy significantly depends on the antibiotic resistance specific to each geographical region; thus, currently, given the increasing prevalence of antimicrobial resistance (especially to clarithromycin, metronidazole, and levofloxacin), successful treatment for Hp eradication has become a real challenge and a critical issue. The most incriminated factors associated with multidrug resistance (MDR) in Hp proved to be the overuse or the improper use of antibiotics, poor medication adherence, and bacterial-related factors including efflux pumps, mutations, and biofilms. Up to 30% of first-line therapy fails due to poor patient compliance, high gastric acidity, or high bacteremia levels. Hence, it is of great importance to consider new eradication regimens such as vonoprazan-containing triple therapies, quintuple therapies, high-dose dual therapies, and standard triple therapies with probiotics, requiring further studies and thorough assessment. Strain susceptibility testing is also necessary for an optimal approach.

## Introduction

### General considerations

*Helicobacter pylori* (Hp) represents a slow-growth Gram-negative helical- or spiral-shaped flagellated bacterium, a urease producer (Bacteriology and Epidemiology of *Helicobacter pylori* Infection–UpToDate, 2022). Infection with Hp is one of the most common chronic bacterial infections, playing an important role in the emergence of chronic progressive gastric inflammation and a variety of gastrointestinal diseases, such as gastric or duodenal ulcers, gastric cancer, or MALT lymphoma (Singh et al., 2017). This pathogen proves strict tropism and is well adapted to the gastric milieu, with specific adhesion to the gastric epithelial cells. Hp colonizes the mucus layer in the gastric antrum or areas of gastric metaplasia in the duodenum, and it requires at least three key characteristics in order to induce the infection: the production of an active urease, the presence of flagella, and the presence of adhesins (Boyanova et al., 2019). Deterioration of the gastric parietal cell is mostly caused by the release of enzymes and the induction of apoptosis by binding to the molecules of the class II major histocompatibility complex (MHC II) (Kusters et al., 2006). The production of bacterial urease allows the conversion of urea into ammonia and chloride, with a direct cytotoxic effect. The passage through the mucus layer to the gastric surface epithelium is made easier by its spiral shape, flagella, and the mucolytic enzymes (Boyanova et al., 2019). Hp attaches through some adhesion molecules, including *BabA*, which, by binding to the Lewis antigen, expressed on the surface of gastric mucosa cells, leads to gastritis among subjects who are infected (Chang et al., 2018). Furthermore, ulcers and gastric cancer appear most frequently when the infectious strain expresses *CagA* (cytotoxic-associated protein) and *VacA* (vacuolating toxin) genes, secondary to an important inflammatory and immune response, mainly associated with the synthesis of interleukin 8 (IL-8), a significant mediator of gastric inflammation (Tshibangu-Kabamba and Yamaoka, 2021). Nevertheless, genetic background is also involved. Thus, even if adhesion is dependent upon the binding of bacterial surface adhesins to specific epithelial cell receptors, host factors could modulate this process (Podolsky et al., 2015). For instance, certain individuals might express specific surface receptors or greater numbers of receptors, making them more susceptible to Hp attachment and colonization. Polymorphisms leading to increased IL-1β levels are associated with atrophic gastritis and cancer (Boyanova et al., 2019).

## Epidemiology

Epidemiologically, Hp affects over 4.4 billion individuals worldwide, with a global incidence of infection reaching up to 50% (Lee, 2019). The prevalence of infection varies between countries, within them, and within the subpopulations of the same country, and it seems that in developing countries, the prevalence is much higher (80–90% of the population) when compared to developed countries (20–50% of the population) (Eusebi et al., 2014; Khoder et al., 2019). It has been demonstrated that the risk of acquiring Hp infection is strongly associated with socio-economic status and hygiene conditions early in life (such as the density of housing, overcrowding, and lack of running water). The highest rate of infection is shown in groups with low socio-economic status and during childhood (Borka Balas et al., 2022). Thereby, those aged between 10 and 19 years from a high socio-economic class had a 20% frequency of Hp infection, while individuals of the same age from a low socio-economic class had a 60% frequency (Hooi, 2017; Talebi and Abadi, 2017). Serologic evidence of Hp is rarely found before age 10 but increases to 10% in those aged between 18 and 30 years and to 50% in those older than 60 years (Lee, 2019). The incidence increases with age, probably due to acquisition during childhood, a period when hygiene is poorer (cohort effect) (Mégraud and Lehours, 2007). Moreover, recent literature highlighted an increased possibility of persistent infection with Hp infection associated with the consumption of salted food (Bacteriology and Epidemiology of *Helicobacter pylori* Infection–UpToDate, 2022).

## Treatment regimens in *H. pylori* infection

Currently available guidelines recommend that all patients with gastric or duodenal ulcers benefit from eradication therapy if Hp is present (Chey et al., 2017). The selection of treatment regimen should be based on local antibiotic resistance patterns (if known), previous exposure and allergies to specific antibiotics, cost, side effects, and ease of administration (Treatment Regimens for *Helicobacter pylori* in Adults—UpToDate, 2022). Good compliance with treatment is highly necessary. In general, eradication regimens consist of two antibiotics administered together with a double dose of proton pump inhibitors (PPIs), to strongly suppress gastric acid secretion (Boyanova et al., 2019). Metronidazole, clarithromycin, amoxicillin, tetracycline, and bismuth represent the pylon agents of the treatment and first-line standard regimens consist of triple therapy (including PPI, clarithromycin, and amoxicillin or metronidazole) for 14 days, concomitant therapy/non-bismuth quadruple therapy (including PPI, clarithromycin, amoxicillin, and a nitroimidazole–tinidazole or metronidazole) for 10 to 14 days, hybrid therapy, as an alternative to clarithromycin triple therapy—consists of PPI and amoxicillin for 7 days followed by PPI, amoxicillin, clarithromycin, and a nitroimidazole for 7 days, and sequential therapy—the 10-day clarithromycin-containing sequential therapy regimen is based on PPI and amoxicillin for 5 days, followed by PPI, clarithromycin, and nitroimidazole (metronidazole) for 5 days (Feldman et al., 2015) (Treatment Regimens for *Helicobacter pylori* in Adults—UpToDate, 2022).

However, the efficacy of Hp eradication treatment has decreased dramatically, and up to 30% of first-line therapy fails (Chey et al., 2017). Several factors, such as poor patient compliance and resistance of Hp strain to several commonly prescribed antibiotics (including vancomycin, trimethoprim, and sulfonamides) or inadequate acid suppression are associated with eradication failure, but the most significant factor incriminated seems to be the increasing regional antibiotic resistance to drugs (Boyanova et al., 2019; Kuo, 2021). It needs to be mentioned that there is a high incidence of resistance to metronidazole and clarithromycin, especially in certain populations, and resistance to clarithromycin has doubled in Europe in the last decade (Boyanova et al., 2019; Megraud, 2021; Treatment Regimens for *Helicobacter pylori* in Adults—UpToDate, 2022). Resistance to amoxicillin, tetracycline, and rifabutin is generally low (< 5%), except in countries where they are available without medical prescription, and resistance could exceed 50% (Treatment Regimens for *Helicobacter pylori* in Adults—UpToDate, 2022). Hence, in patients who have failed standard therapy, salvage regimens should be considered: bismuth quadruple therapy (including PPI, tetracycline, metronidazole, and bismuth subsalicylate), levofloxacin-based therapy triple (including PPI, levofloxacin, and amoxicillin), or quadruple (including PPI, levofloxacin, nitazoxanide, and doxycycline)—other levofloxacin-based quadruple therapies include PBLA (PPI, bismuth, levofloxacin, and amoxicillin), PBLT (PPI, bismuth, levofloxacin, and tetracycline), and PBLM (PPI, bismuth, levofloxacin, and metronidazole) (Shah et al., 2021), high-dose dual therapy (including PPI and amoxicillin—at least 2 g divided three or four times per day to avoid low trough levels) for 14 days—particularly in patients in whom dual metronidazole/clarithromycin resistance or levofloxacin resistance is suspected, rifabutin triple therapy (including PPI two times daily, rifabutin and amoxicillin) for 14 days, and clarithromycin-based therapy (including PPI, bismuth, clarithromycin, and tetracycline) (Chey et al., 2017; Treatment Regimens for *Helicobacter pylori* in Adults—UpToDate, 2022). It should be highlighted that levofloxacin should be used only if the Hp strain is known to be sensitive to it or if the population levofloxacin resistance rates are <15%, taking into account that levofloxacin resistance decreases the eradication success rate of levofloxacin-containing regimens by 20–40% (Karamanolis et al., 2014; Lee, 2019; Lee et al., 2019). Regarding clarithromycin-based therapy, it could only be used as a salvage regimen in patients with no risk factors for macrolide resistance (no prior macrolide exposure and local clarithromycin resistance known to be <15%) (Talebi and Abadi, 2017; Lee, 2019). Moreover, rifabutin-based triple therapy is not only expensive but also could lead to reversible myelotoxicity and could increase the prevalence of rifabutin-resistant mycobacteria (Lee et al., 2019). According to The Maastricht V/Florence consensus, the first-line treatment regimen is given considering the resistance to clarithromycin, determined by antibiogram cultures or molecular tests, with a threshold of 15% (Malfertheiner et al., 2017). In regions with increased resistance to clarithromycin, bismuth quadruple therapy is recommended as the first-line therapy, and if not available, sequential therapy or quadruple therapy is recommended. Furthermore, to amplify the efficiency of both standard therapy and salvage regimens, the latest Maastricht V consensus recommends increasing the duration of treatment administration from 7 to 14 days and the usage of a double dose of PPI compared to the last recommendation, acquiring higher eradication rates with 5% and 8–12%, respectively (Malfertheiner et al., 2017).

## Multidrug resistance in *Helicobacter pylori*

**Figure 1:**
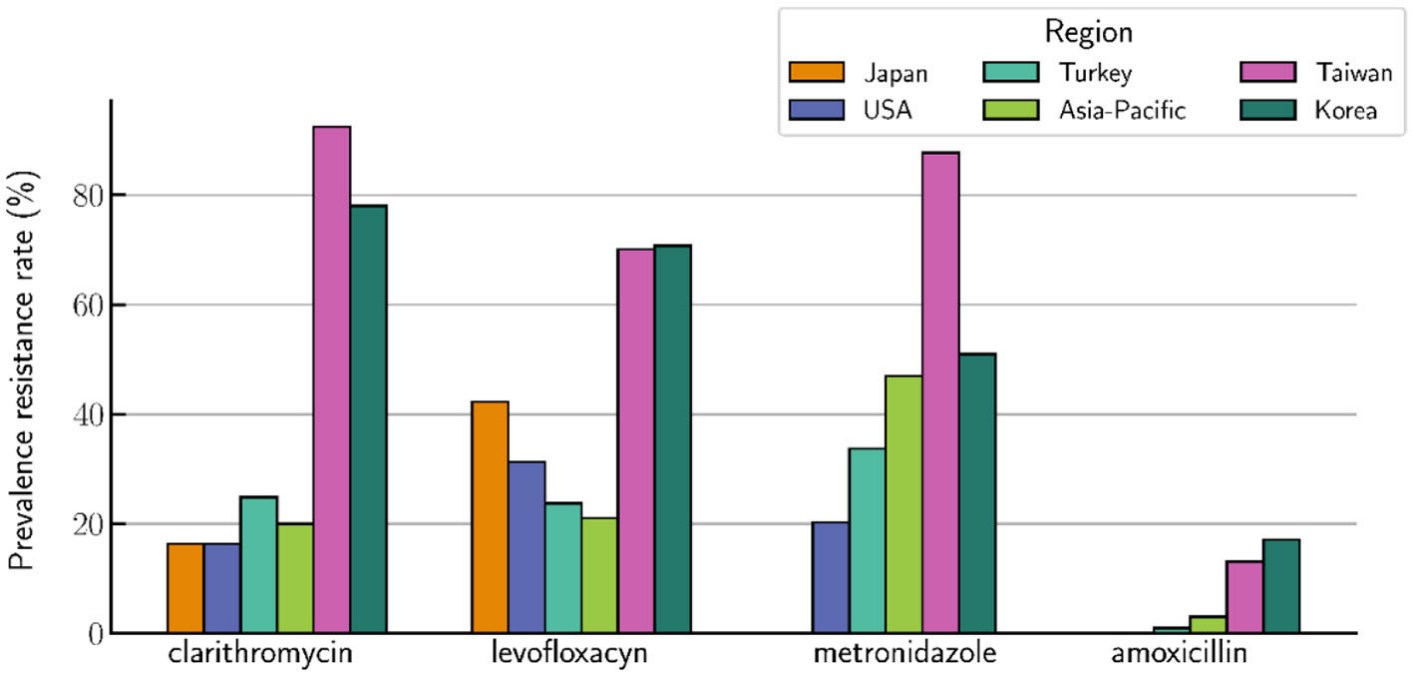
Prevalence of MDR rate (%) by region.

Worldwide, an alarming and substantial concern arises from multidrug resistance (MDR) in Hp, leading to therapeutic regimen failures and low eradication rates. MDR is defined as resistance to ≥3 antibiotics of different classes and it depends on the geographical area, study period, and patients' characteristics (Boyanova et al., 2019; Sukri, 2022) (Figure 1).

### Factors associated with MDR

Taking into consideration the widespread and improper use of antibiotics, one crucial factor influencing MDR in Hp is national outpatient antibiotic consumption; thus, one study assessing the primary Hp resistance in 18 European countries revealed that outpatient antibiotic consumption influenced both macrolide and fluoroquinolone resistance (Megraud, 2013). Furthermore, Hp efflux pumps and biofilms (multidimensional matrix-enclosed bacterial communities linked to chronic infections and reduced antibiotic susceptibility) seem to be involved in MDR (Singh et al., 2017). Not only were enhanced expression of efflux pump (*hp1165* and *hefA*) genes involved in tetracycline and MDR resistance noticed in Hp biofilms but also the upregulation of genes four transmembrane ABC transporters and other (hp0656, hp0946) efflux proteins were detected (Attaran et al., 2017; Kazakos et al., 2017). In contrast, resistance could be undetected when low-density inoculum is used for susceptibility testing, in case of mixed infections which could carry both susceptible and resistant isolates to an antibiotic (Mansour, 2016; Lee, 2019). Last but not least, poor adherence to current guidelines for the management of Hp infection could lead to the occurrence of MDR strains and treatment failure.

### Single-drug resistance mechanisms

Clarithromycin is a macrolide antibiotic whose action is based on the interaction with the peptidyl transferase loop of the V domain of the 23S ribosomal RNA molecule, which could inhibit bacterial protein synthesis (Talebi and Abadi, 2017). Hence, point mutations in the V domain of the 23S ribosomal RNA might inhibit the affinity between clarithromycin and the peptidyl transferase loop, leading to clarithromycin resistance (Stone, 1996; Talebi and Abadi, 2017). Among responsible mutations, the 23S ribosomal RNA A2143G, A2142G, and A2142C were reported to be the most frequent, accounting for up to 90% (Mégraud and Lehours, 2007). Another potential mechanism incriminated in clarithromycin resistance of Hp is represented by the efflux pump system, and the currently available data suggest that the existence of efflux pumps in Hp strains could have a synergic effect to induce antibiotic resistance in parallel with 23S rRNA mutations (Talebi and Abadi, 2017). In contrast, one study suggested that the outer membrane protein (OMP) alterations might be involved in the Hp resistance to clarithromycin. By using comparative proteomic analyses of clarithromycin-susceptible and -resistant Hp strains to identify Hp OMPs, Smiley et al. reported that iron-regulated membrane protein, urease B, elongation factor thermo unstable, and putative OMP were downregulated, whereas HopT (BabB) transmembrane protein, HofC, and OMP31 were upregulated in clarithromycin-resistant Hp (Smiley et al., 2013).

Metronidazole is a synthetic nitroimidazole, representing one of the mainstay drugs for the treatment of anaerobic infections. Concerning the mechanisms responsible for resistance to metronidazole in Hp, data highlighted mutations of *rdxA*, a gene that encodes an oxygen-insensitive NADPH nitroreductase, as being the main cause (Kim, 2009; Lee, 2018). Furthermore, mutations in other redox genes such as *frxA* (encoding the NADPH flavin oxidoreductase) and *fdxB* (encoding the ferredoxin-like protein) might also induce Hp resistance to metronidazole (Saranathan, 2020; Metronidazole: An overview–UpToDate, 2022). In contrast, in one study, Mehrabadi et al. suggested that the resistance nodulation cell division (RND) family of efflux pumps might be involved in the metronidazole resistance of Hp clinical isolates and they reported that excess amounts of metronidazole increased the gene expression levels of the outer membrane protein (TolC) homologs of RND pumps (Mehrabadi et al., 2010; Lee, 2018).

Levofloxacin represents a fluoroquinolone drug that exerts its antibacterial effect through the interaction with DNA gyrase, encoded by *gyrA* and *gyrB*. Thus, point mutations in the quinolone resistance-determining regions of *gyrA* might restrict this process, leading to fluoroquinolone resistance of Hp (Tankovic et al., 2003). Rimbara et al. indicated that a *gyrB* mutation at position 463 might also induce Hp resistance to fluoroquinolone (Rimbara et al., 2012). The literature shows that mutations in 87, 88, 91, and 97 positions of *gyrA* are the most common (Rimbara et al., 2012; Shetty et al., 2019; Keikha et al., 2022).

Amoxicillin, one of the most commonly used antibiotics in the primary care setting, is a beta-lactamase antibiotic that interacts with penicillin-binding proteins (PBPs) and inhibits the synthesis of the cell wall, resulting in bacterial dissolution (Akhavan et al., 2022). Evidence highlighted that high levels of amoxicillin resistance are associated with the production of beta-lactamase in Hp (Sukri, 2022). Decreased membrane permeability to amoxicillin or the alteration of the efflux pump might also be involved. In addition, it seems that the most common mechanism that contributes to low or moderate levels of amoxicillin resistance is represented by point mutations in the *pbp 1A* gene, but mutations in the *pbp 2, pbp 3, hefC, hopC*, and *hofH* were also mentioned in the literature (Okamoto, 2002; Qureshi et al., 2014).

Tetracycline is a macrolide antibiotic that inhibits protein synthesis by blocking the attachment of charged tRNA at the P site peptide chain. Tetracycline binds to the 30S and 50S subunits of microbial ribosomes and bacteria usually acquire resistance from the horizontal transfer of a gene that either encodes an efflux pump or a ribosomal protection protein (Grossman, 2016). Data reported that single and double-base-pair mutations were only responsible for low levels of tetracycline resistance, while triple-base-pair mutations *16S rDNA AGA (926–928) were* associated with high levels of resistance (Gerrits et al., 2003). Moreover, in one study, Anoushiravani et al. suggested that proton motive force-dependent efflux mechanisms might be involved in the resistance of Hp clinical isolates to tetracycline (Anoushiravani et al., 2009).

### Multidrug resistance mechanisms

As mentioned earlier, MDR is defined as resistance to ≥3 antibiotics of different classes, and the increasing presence of Hp strains with an MDR profile represents a serious threat globally (Boyanova et al., 2019). Despite various mutations that simultaneously induce resistance to separate drug families conferring a cumulative MDR profile, there are additional mechanisms responsible for MDR in Hp, but data are still limited (Tuan, 2019). For instance, Hp could turn into quiescent cells named coccoid forms for which substantially increased minimum inhibitory concentrations of different antibiotics are required to achieve bactericidal effects (Kadkhodaei et al., 2020). Hence, considering subsequent ultrastructural modifications in the cell membrane and metabolic pathways that reduce drug target exposure and drug penetration, the coccoid formation could be a leading cause of MDR (Kadkhodaei et al., 2020). In addition, biofilm formation could play an important role in antibiotic resistance, yet the mechanism is not completely understood (Hathroubi et al., 2018). Furthermore, even though the information is limited, studies reported that the upregulation of efflux pump systems against a corresponding group of substrates and restricted drug uptake owing to the downregulation of expression of outer membrane proteins or lipopolysaccharides could also be involved in acquiring MDR in Hp (Bos et al., 2004; Hirata, 2010; Ge, 2018) (Table 1).

**Table 1:** Biological mechanisms, molecular target, and gene or sequence name of drug resistance in *Helicobacter pylori* species reported following standard recommendations in molecular diagnostics from the Human Genome Variation Society (Den Dunnen and Antonarakis, 2001; Kusters et al., 2006; Ogino, 2007; Chang et al., 2018).

**Antibiotic**	**Resistance mechanism**	**Molecular target**	**Gene or sequence name**
Clarithromycin	Protection of the mRNA–tRNA translocation step during protein synthesis	23S rRNA with a V domain altered by base-pair substitutions	*23S rRNA, rpl22, infB*
	Putative protection of ribosomal domains	Rpl22 or InfB with alterations by missense mutations, or indels	
Metronidazole	Reduced or suppressed drug reductive activation by altered oxygen-insensitive nitroreductases	FrxA and/or RdxA with altered molecule stability, dimerization, or flavin mononucleotide binding by frameshift, nonsense, indel, or missense mutations	*rdxA*-related and *rdxA*-related promoter region, *frxA, fur, sodB*-related promoter region, *recA, mdaB, ribF, omp11, rpsU*
	Reduced or suppressed drug reductive activation by downregulated oxygen-insensitive nitroreductases	Downregulated expression of RdxA probably by mutations in related promoter region	
	Regeneration of inactive drug compounds by increased futile cycling of oxygen and drug; protection against oxidative reactions	Hyperactivity of oxygen “futile cycle”; upregulation of SodB by inactivation of Fur activity due to Fur missense or nonsense mutation, and single, base-pair substitution in sodB promoter region	
	Protection of DNA from damage by ROS and toxic drug derivatives	Upregulation of RecA DNA repair effector due to missense mutations	
Levofloxacin	Protection of chromosomal supercoiling during DNA synthesis, transcription, and cell division	GyrA and GyrB with sequence alterations inside or outside QRDR by missense mutations	*gyrA, gyrB*
Amoxicillin	Protection of peptidoglycan synthesis during cell wall synthesis	PBPs (i.e., PBP1A, PBP2, PBP3); alteration by missense, indel or nonsense mutations in or around PBP motifs (SxN, KTG, and SxxK) and PBP C terminus sequences	*pbp-1A, pbp2, pbp3, pbp4, hofH, hefC, hopC*
	Putative decrease in membrane permeability to the drug	HopC and HofH porins; alteration by missense mutations	
Tetracycline	Protection of the peptide chain elongation step during protein synthesis	16S rRNA with a tetracycline-binding pocket altered by single, double, or triple base-pair substitutions	*16S rRNA*

### Heteroresistance mechanisms

Heteroresistance consists of the presence of a heterogeneous population of bacteria with one subpopulation or several subpopulations that exhibit increased levels of antibiotic resistance compared to the main population and could be considered a proclaimer of single-drug resistance or MDR (Andersson et al., 2019). Monoclonal or polyclonal differentiation of bacterial population represents the main mechanisms responsible for this phenomenon (Ailloud, 2019; Andersson et al., 2019). It seems that anatomical and physiological differences between the antral and oxyntic gastric mucosa compose an evolutionary force that transduces intragastric migrations of bacteria from the same clone and rapid adaptation to microniches within the host; therefore, the population structure of bacteria could be partitioned through evolutionary bottlenecks in subgroups (Ailloud, 2019). Concerning Hp, it is more probable that heteroresistance is determined by the same strain with and without resistance attributes rather than a co-infection with different strains (Ailloud, 2019). Studies suggest that multiple gastric biopsy specimens or multiple bacterial colonies from the same sample should be obtained for drug susceptibility testing so as to counteract this phenomenon (Kao, 2014; Andersson et al., 2019).

### Prevalence of MDR

The prevalence of MDR is characterized by a regional variation and appears to be changing over time. Literature reported lower rates of primary MDR, ranging from ≤10% in Austria, Portugal, Argentina, France, and Bulgaria to >20% in India and >40% in Peru, while in treated patients, MDR rates were >16%, reaching 31.6% in Vietnam (Almeida, 2014; Phan, 2015; Mansour, 2016; Zollner-Schwetz, 2016; Boehnke, 2017; Boyanova, 2017). In one meta-analysis evaluating the primary antibiotic resistance in the Asia-Pacific region between 2006 and 2015, Kuo et al. reported a primary resistance to clarithromycin, metronidazole, and levofloxacin of 20%, 47%, and 21%, respectively (Kuo, 2017; Savoldi et al., 2018). Both tetracycline and amoxicillin primary resistance rates were 3%, and the amoxicillin primary resistance rate was relatively low, up to 5% in the majority of countries (Lee et al., 2019). The prevalence of primary phenotypic resistance to levofloxacin was >10% in France (17.2%), Belgium (16.8%), Japan (15%), Hong Kong (11.5%), and Korea (10.4%), but <10% in Taiwan (5.8%) (Kuo, 2017). Concerning secondary resistance rates, one study reported secondary resistance rates to amoxicillin, clarithromycin, metronidazole, tetracycline, and levofloxacin of 13.1, 92.5, 87.7, 14.3, and 70.1%, respectively, in patients who had received these antibiotics in their prior therapies in Taiwan (Liou, 2013). In addition, one recent study conducted in Korea reported secondary resistance rates to amoxicillin, clarithromycin, metronidazole, tetracycline, and levofloxacin of 17.1, 78.0, 51.2, 12.2, and 70.7%, respectively (Lee et al., 2019). There are also variations in MDR rates among regions in the same country (Savoldi et al., 2018). For instance, in Korea, MDR prevalence was <20% in Seoul and Chungcheong and >30% in Cholla and Gyeonggi (Lee, 2019).

Most frequently, MDR in Hp is triple or quadruple, though several countries, such as China or Bulgaria, reported quintuple resistance (Zhang et al., 2015; Boyanova, 2017). For instance, in Spain, triple resistance altered from 3.3% in 2013 to 1.8% in 2015 and 2.4% in 2017 (Cosme, 2019). In Bulgaria, the quintuple Hp resistance proved to be to amoxicillin, metronidazole, clarithromycin, tetracycline, and levofloxacin; while in China, the quintuple resistance proved to be to clarithromycin, metronidazole, levofloxacin, tetracycline, and rifampicin or amoxicillin, clarithromycin, metronidazole, levofloxacin, and rifampicin or amoxicillin, clarithromycin, metronidazole, levofloxacin, and tetracycline; in Chinese patients, triple, quadruple, and quintuple resistance rates were 24.9, 7.3, and 2.3%, respectively (Zhang et al., 2015; Boyanova, 2017). Moreover, in China, while the resistance rates to amoxicillin and tetracycline have remained relatively stable and low when it comes to clarithromycin, metronidazole, and levofloxacin, the resistance rates have increased considerably; thus, the eradication rate of standard triple therapies has gradually decreased from 88.54% (pre-2004) to 77.66% (2005–2009) and 71.13% (2010–2013) (Wang, 2014; Zhang et al., 2015). Consistently, in Japan, studies reported high rates of clarithromycin and levofloxacin resistance (16.4–81.1% and 42.3–43.2%, respectively), and low rates of amoxicillin resistance (~0.03%) (Nishizawa et al., 2015; Sugimoto, 2017). Moreover, an increasing trend of resistance to clarithromycin, levofloxacin, and metronidazole was observed in the USA, as well. Hence, in a group of veteran patients, Shiota et al. reported resistance rates of 31.3, 20.3, and 16.4% to levofloxacin, metronidazole, and clarithromycin, respectively, with an increase in clarithromycin resistance from 9.1% on 2009–2010 to 24.2% during 2011–2013 (Shiota et al., 2015). Multiple single-center studies conducted in Poland, Greece, and Germany showed high clarithromycin resistance in Poland and Greece and increased metronidazole resistance in Poland and Germany (Karamanolis et al., 2014; Karpiński et al., 2015; Regnath et al., 2017). In Turkey, Kocazeybek et al. reported resistance rates of 24.86, 33.75, 23.77, 3.51, and 0.97% for clarithromycin, metronidazole, levofloxacin, tetracycline and amoxicillin, respectively (Kocazeybek and Tokman, 2016). In contrast to the rising tendency of Hp multi-resistance to antibiotics observed in most European countries, Mourad-Baars et al. reported remarkably low rates of Hp antibiotic resistance in the Netherlands, particularly for clarithromycin and metronidazole (Mourad-Baars et al., 2015).

Unfortunately, for now, there are not sufficiently large epidemiological studies in Romania to evaluate the level of resistance to clarithromycin; however, metronidazole has shown an increased resistance rate *in vitro*, over 90%, most probably due to overuse for other infections.

### Clinical implications of MDR

The main clinical implication of bacterial resistance *in vitro* is a substantial decrease in the efficacy of the Hp treatment—an outcome that has been previously outlined as well. The theoretical consequence is an associated increase in clinical complications such as gastric cancer or peptic ulcers due to the longer duration of infections (Tshibangu-Kabamba and Yamaoka, 2021). The treatment efficacy is majorly impacted primarily by pretreatment antibiotic resistance according to studies that mainly assessed the effect of single-drug resistance to clarithromycin, metronidazole, and levofloxacin (Kasahun et al., 2020; Zou, 2020). Dramatic reductions in treatment efficacy have been found in the case of single-drug resistance to clarithromycin during triple and quadruple therapies containing clarithromycin (Dore et al., 2000; Zou, 2020). Lower decreases in the treatment success rate have been found for metronidazole resistance with triple and non-bismuth quadruple therapies. Nevertheless, even in the presence of bacterial resistance, a very high therapeutic efficacy was reported with bismuth quadruple therapies (Fischbach and Evans, 2007; Zou, 2020). Further evidence is needed to evaluate the effect of single-drug resistance to levofloxacin (Zou, 2020). Overall, an increasing pattern of single-drug resistance in Hp has been noted worldwide which could be associated with an increase in the rate of treatment failure (Kasahun et al., 2020).

The clinical implications become even more challenging in the case of MDR and heteroresistance in Hp when compared to that of single-drug resistance due to the multiple drug molecules that are affected simultaneously in the former case (Andersson et al., 2019). However, despite the clinical challenges, there is evidence of Hp eradication being attainable in patients (Fischbach and Evans, 2007). The effectiveness of the eradication therapy was suggested to also depend on factors related to the host rather than only on considerations of the pathogen's biology (Chey et al., 2017). For instance, several factors that dictate the extent to which bacterial resistance affects the efficacy of a given therapy are represented by the dose of antimicrobial agents, the duration of therapy, and the components used in therapy (Graham et al., 2014; Smith et al., 2014). Moreover, the interplay between the drug resistance of Hp and other microbial species is also of high concern for human health. It has been demonstrated that, despite inducing gastrointestinal dysbiosis, Hp infection and the associated eradication therapy could significantly increase MDR and single-drug resistance mechanisms in other microbial species through its drug resistance and eradication failure (Chen, 2018; Iino, 2018; Wu et al., 2019; Guo, 2020). Based on these findings, the successful eradication of Hp translates into long-term benefits for other microbial drug resistance mechanisms.

### Future directions

The changing profile of Hp antibiotic resistance has reached alarming levels worldwide, with a great impact on the efficacy of empirical therapies. Despite the appropriate eradication regimens, it seems that about 10% of patients might have a persistent infection; hence, further development and discovery of novel regimens and approaches against Hp infection are needed (Siddique et al., 2018).

In an era of increasing MDR, classic bismuth-containing quadruple therapies (BQTs) consisting of bismuth, a PPI, metronidazole, and tetracycline remain the central pylon and are recommended as first-line treatments for Hp infections. In a review focused on the role of bismuth in improving Hp eradication with triple therapies, Graham and Dore suggested that bismuth in addition to a 14-day triple therapy might improve eradication rates with almost 40%, even in a high prevalence of antimicrobial resistance (Graham and Dore, 2016). In addition, taking into account that most eradication regimens are not efficient for treating MDR infections, currently, bismuth-containing quadruple therapy is strongly recommended in regions with high Hp resistance to both clarithromycin and metronidazole, according to Maastricht V consensus (Malfertheiner et al., 2017). A combination of the three-in-one capsules (metronidazole 125 mg, tetracycline 125 mg, and bismuth subcitrate 140 mg) named Pylera has been approved by the United States Food and Drug Administration (FDA) (Treatment Regimens for *Helicobacter pylori* in Adults—UpToDate, 2022). Pylera could be used in combination with double-dose PPIs and the literature highlighted that eradication success of PPI/Pylera therapy was ≥90% in infections with metronidazole-resistant strains as well as second-line therapy following clarithromycin-based regimens, regardless of PPI dose or type (Nyssen et al., 2019).

Furthermore, maintaining the gastric microenvironment at a pH > 6 represents a key factor in Hp eradication, and the main role of PPIs in the treatment of Hp infection is to elevate the gastric pH. Gene polymorphisms of the principal enzyme implicated in the metabolism of PPIs, CYP2C19, have a major implication in the efficacy of Hp eradication, and literature showed that in CYP2C19 extensive metabolizers, second-generation PPIs such as esomeprazole and rabeprazole provided better eradication rates than first-generation PPIs including omeprazole, pantoprazole, and lansoprazole (McNicholl et al., 2012; Padol et al., 2012). In one study comparing the acid-inhibitory effects of four PPIs (omeprazole, lansoprazole, esomeprazole, and rabeprazole), Sahara et al. reported median pH levels of 5.0, 4.7, 5.4, and 4.8 after treatment with omeprazole, lansoprazole, esomeprazole, and rabeprazole, respectively. The study showed that 20 mg of esomeprazole dosed two times daily provided the strongest inhibition in rapid CYP2C19 metabolizers (Sahara, 2013). In addition, evaluating the effect of CYP2C19 polymorphisms on the efficiency of Hp eradication, literature indicated esomeprazole or rabeprazole as being the less influenced PPIs by CYP2C19 polymorphisms recommended in Hp eradication regimens, particularly in areas with a high proportion of rapid metabolizers, such as Europe and North America (Malfertheiner et al., 2017).

A novel potassium-competitive acid blocker, vonoprazan, is reported to provide better efficacy, safety profile, and prolonged activity when compared to other PPIs, due to the capacity to accumulate in high concentrations and to be slowly cleared from gastric glands, increasing gastric pH levels and producing more potent and sustained acid-inhibitory effects (Graham, 2017). For instance, in Japan, vonoprazan proved higher eradication rates, approximately 90%, in triple therapy with amoxicillin and clarithromycin or metronidazole (Tanabe, 2017; Furuta, 2020). Moreover, in a randomized, double-blind, multicenter, parallel-group study, Murakami et al. reported an eradication rate of Hp of 92.6 vs. 75.9% with lansoprazole (Murakami et al., 2016). In the clarithromycin-susceptible strain subpopulation, a vonoprazan triple therapy was not superior to a lansoprazole triple therapy, with eradication rates being 97.6 vs. 97.3%; whereas in the subpopulation with clarithromycin-resistant strains, it was noticed a higher eradication rate with the vonoprazan triple therapy than the lansoprazole triple therapy, 82.0 vs. 40.0%, both the first-line and second-line vonoprazan triple therapies being well-tolerated. However, a cure rate of 82% is still low; hence, further studies concerning the efficacy of vonoprazan-based therapies with higher doses and longer durations are required (Murakami et al., 2016; Graham, 2017).

New antibacterial agents such as delafloxacin (a newer fluoroquinolone) or flavodoxin inhibitors (7-nitrobenzoxadiazole derivatives) are also mentioned in the literature, but further research regarding their effectiveness and safety is needed.

Antibiotic adjuvants such as probiotics (Pbs) have great potential, considering the ability to raise antibiotic activity and inhibit several resistance mechanisms by immune modulation, producing antioxidants and antimicrobial substances, altering local pH, or affecting Hp colonization and adherence to gastric cells (Ruggiero, 2014; González-Bello, 2017). Numerous meta-analyses highlighted the significant role of Pbs, mainly *Lactobacillus* or *Saccharomyces boulardii* or *Bacillus clausii*, in improving the eradication rate of Hp. In one study, McFarland et al. reported high eradication rates, >90%, using four probiotic mixtures (*L. acidophilus/B. animalis, L. helveticus/L. rhamnosus, L. acidophilus/B. longum/E. faecalis* and the eight-strain mixture) as adjuvants for Hp eradication, for long duration (3–5 weeks) and at high doses (McFarland et al., 2016). Another meta-analysis found that patients treated with Pbs had a higher eradication rate than those not treated with Pbs (80.3 vs. 72.2%) (Lv, 2015). It seems that Pbs might significantly reduce the adverse effects of the treatment regimens, and the administration before or after the eradication treatment for >2 weeks could improve the eradication success (Lv, 2015).

Recently, the new concept of the anti-biofilm approach has gained interest, but information about the molecular mechanisms which lead to Hp biofilm formation is still deficient. However, two synthetic anti-biofilm peptides, IDR-1018 and DJK-5, were discovered, which are active only against the bacteria without affecting the planktonic *H. pylori* and affect different biofilm formation stages (Windham et al., 2018). Moreover, in one study, adding a glycolipid biosurfactant, rhamnolipid, raised the anti-biofilm activity of the treatment and the combination of rhamnolipid, PPI, and amoxicillin conducted to 95% biofilm eradication (Chen et al., 2019).

Another potential perspective consists in anti-Hp vaccines, which could be prophylactic, by preventing initial Hp colonization, or therapeutic, as a possible alternative of or adjunct to the eradication therapy (Talebi and Abadi, 2016). One randomized placebo-controlled trial in phase III reported high rates of vaccination success (71.8%) with an anti-Hp oral recombinant vaccine in Chinese children within the first year (Zeng et al., 2015). The development of effective vaccines could represent a powerful strategy to reduce the prevalence of Hp infection and for eradication failure, but it requires epitope mapping, choice of antigen determinants, genomic approach, and safety assessment (Mirzaei et al., 2017).

Last but not least, it is of great importance to perform strain susceptibility testing. One study reported successful eradication of 94% and fewer adverse effects (15%) when using antibiotic susceptibility guided regimens vs. eradication success of 87% with empirical concomitant therapy with more frequent adverse effects (31%), supporting the major impact of culture-guided treatment (Cosme et al., 2016).

## Conclusion

The alarming increase of MDR in Hp infection, leading to eradication failure, represents a serious challenge, and addressing this global problem implies not only novel antimicrobial drugs and treatment strategies but also improved diagnostic tools to guide clinicians in further optimizing currently available regimens. Hence, strict adherence to current guidelines and a complex multidisciplinary approach for improving the standard treatment regimens or developing novel strategies in the battle against Hp MDR is highly necessary.

## Author contributions

RD, MM, AS, and OA contributed to conception and design of the study. OA organized the database. DP and AC wrote the first draft of the manuscript. RD, AC, AS, and OA wrote sections of the manuscript. All authors have contributed equally in writing, reading, and approving the final manuscript.
